# Optimized *ex vivo* expansion and functional characterization of canine natural killer cells using genetically modified feeder cells

**DOI:** 10.4142/jvs.24345

**Published:** 2026-06-19

**Authors:** Yurim An, Geun-Seop Kim, YeonJu Baek, Jae-Hee Park, Jeong Won Hong, Phuoc Quang Huy Nguyen, Sang-Ki Kim

**Affiliations:** 1 Department of Applied Biotechnology, College of Industrial Science, Kongju National University, Yesan 32439, Korea.; 2 Department of Companion and Laboratory Animal Science, College of Industrial Science, Kongju National University, Yesan 32439, Korea.; 3 Research Institute for Natural Products, Kongju National University, Yesan 32439, Korea.; 4 Vaxcell-bio, Co., LTD., Hwasun 58128, Korea.

**Keywords:** Dogs, immunotherapy, adoptive, cell manufacturing, cytotoxicity, immunologic, ARH77 cells

## Abstract

**Importance:**

Natural killer (NK) cells are critical effectors of innate immune surveillance and can eliminate malignant cells without prior sensitization. Given the biological and genetic parallels between canine and human cancers, NK cell-based immunotherapies have strong translational potential in veterinary oncology. However, the lack of an efficient and reproducible *ex vivo* expansion protocol for canine NK cells remains a major barrier to clinical application. Establishing culture conditions for expansion of functional canine NK cells would support adoptive NK cell therapy and translational research.

**Objective:**

This study aimed to evaluate the effectiveness of genetically modified feeder cells and optimized culture media in expanding canine NK cells.

**Methods:**

Canine peripheral blood mononuclear cells were co-cultured with irradiated genetically modified K562 or ARH77 cells in either RPMI-1640 or Dulbecco’s Modified Eagle Medium (DMEM)/F12-based media. NK cell expansion, purity, cytotoxicity and expression of NK cell-associated receptors were evaluated using flow cytometry, cytotoxicity assays, and quantitative reverse transcription polymerase chain reaction.

**Results:**

The combination of DMEM/F12-based medium and genetically modified ARH77 feeder cells yielded the highest NK cell expansion rate and purity among the tested conditions. Cytotoxicity differed significantly according to medium type, whereas no significant difference was observed between feeder cell types. In addition, the NK cell-associated receptors were significantly upregulated in expanded NK cells.

**Conclusions and Relevance:**

Genetically modified ARH77 feeder cells with DMEM/F12-based culture media demonstrated superior efficiency in promoting the *ex vivo* expansion of canine NK cells, achieving high purity and enhanced cytotoxic potential. These findings highlight a promising and reproducible platform for generating functional NK cells, offering a practical and scalable strategy to support the development of NK cell-based veterinary immunotherapy.

## INTRODUCTION

Natural killer (NK) cells are a type of innate immune cells that exhibit natural cytotoxicity against cancer cells [1,2]. In addition, they can be used in allogeneic hematopoietic stem cell transplantation or donor lymphocyte infusion, indicating their potential effectiveness as cell therapy agents [3]. However, NK cells consist of only a small proportion of peripheral blood mononuclear cells (PBMCs), posing a challenge in obtaining sufficient NK cells to fulfill clinical requirements that demand a large quantity of infusions [4]. Improving the technology used to efficiently culture and purify NK cells with effective anticancer capacity *ex vivo* is an important step in advancing adoptive transfer therapy [3,5,6]. To selectively amplify NK cells from PBMCs, cytokines such as IL-2 and IL-15 can be used [7,8] and co-cultured with feeder cell lines [9]. Among them, K562 cells transfected with the membrane-bound IL-15 and 41BBL genes (K562-membrane-bound IL-15-41BBL) [10,11] and K562 cells transfected with the OX40 gene (K562-OX40) [12] are known to be more effective in stimulating NK cells when co-cultured with PBMCs than K562 cells without the gene. When culturing human NK cells, it is evident that a medium containing Dulbecco’s Modified Eagle Medium (DMEM)/F12 is more effective in amplifying NK cells compared to that of RPMI-1640 medium [13].

Due to the extended lifespan of dogs resulting from advancements in scientific nutrition, appropriate treatment, and vaccinations, a significant number of cancer cases have been identified in dogs, leading to a growing interest in various treatments [14,15]. Like humans, dogs develop various types of cancer including lymphomas, melanomas, and mammary tumors, exhibiting remarkable similarities in tumor biology, genetics, and treatment response [16,17]. However, the drugs used to treat dogs are often the same as those used to treat human patients, and because they are used at lower dose than those used in humans, the cure rates are lower treated with the same drugs. Therefore, there is a need for research on cancer drugs optimized for dogs [14,18]. Consequently, cellular therapies, which are popular in the medical field, are gaining attention in the veterinary field for cancer treatment in dogs, propelled by persistent constraints in veterinary oncology (e.g., limited species-specific reagents and incomplete NK-cell phenotypic definition) and by the recognized One Health translational value of spontaneous canine cancers; accordingly, NK-cell–based platforms optimized for dogs are emerging as priority avenues and are being actively explored in clinical setting [19,20,21]. Thus, identifying the optimal conditions for *ex vivo* expansion of NK cells in dogs is critical for advancing their potential in cancer immunotherapy. Therefore, this study aims to establish optimal ex vivo expansion conditions by comparing the proliferation capacity, purity, and cytotoxicity of canine NK cells expanded using genetically engineered K562 and ARH-77 feeder cells in different culture media. The effectiveness of each feeder cell type in conjunction with RPMI-1640- and DMEM/F12-based media to promote *ex vivo* expansion of canine NK cells will be assessed [22]. In addition, the expression levels of NK cell receptors in various media and feeder cell combinations will be analyzed to provide a comprehensive understanding of the factors that influence NK cell activation and expansion.

## METHODS

### Cell lines and cytokines

The genetically modified K562 cells expressing membrane-bound CD137L and IL-15 (CD137L-IL-15; GE-K562, K) and ARH77 cells expressing membrane-bound B7H6, CD137L, IL-15, and IL-15 receptor alpha (IL-15Rα) (B7H6-CD137L-IL-15-IL-15Rα; GE-ARH77, A) were cultured in a humidified 5% CO_2_ incubator at 37°C using RPMI-1640 medium supplemented with 10% heat-inactivated fetal bovine serum (FBS), 100 U/mL of penicillin and 100 μg/mL of streptomycin (all from WELGENE, Korea). Canine thyroid adenocarcinoma (CTAC) cells were obtained from the European Collection of Cell Cultures (Salisbury, UK) and cultured in RPMI-1640 medium supplemented with 10% FBS, 100 U/mL of penicillin, 100 μg/mL of streptomycin and 4 mmol/L of L-glutamine (WELGENE) at 37°C in a humidified 5% CO_2_ incubator. Recombinant human IL-2 (PeproTech, USA), recombinant canine IL-15 (in-house) and IL-21 (R&D Systems, USA) were used to expand canine NK cells.

### Isolation of canine peripheral blood mononuclear cells (PBMCs) and expansion of canine NK cells

The use of animals for this study was approved by Institutional Animal Care and Use Committee of Kongju National University (KNU_2024-09). Blood was collected from healthy mixed-breed dogs (male and female; neutered and intact; aged 2–8 years) at 10–15 mL/kg of body weight with the consent of their owners. Whole blood was collected in Vacutainer ACD Solution A tubes (Becton Dickinson, USA). Diluted blood was centrifuged at 400 g for 25 min and separated by density using Histopaque-1119 (Sigma-Aldrich, USA) and Lymphoprep solution (PROGEN, Germany), and washed twice with Dulbecco’s Phosphate-Buffered Saline (WELLGEN). Isolated canine PBMCs were co-cultured with GE-K562 cells or GE-ARH77 cells irradiated with 125 Gy gamma ray in 24-well plates. The culture media used were either RPMI-1640 (WELGENE) supplemented with 10% FBS, 100 U/mL penicillin, 100 μg/mL streptomycin, and 4 mmol/L l-glutamine (RPMI medium; RPMI), or a mixture of DMEM (GIBCO, USA) and Ham's F-12 Nutrient Mix (GIBCO) at a 2:1 ratio (DMEM/F12 medium; D/F12). The DMEM/F12 medium was further supplemented with 10% FBS (WELGENE), 1X glutamax (GIBCO), 25 μM β-mercaptoethanol (GIBCO), 10 μg/mL gentamicin (GIBCO), 50 μM ethanolamine (Sigma-Aldrich), 20 μg/mL ascorbic acid (Sigma-Aldrich), and 5 ng/mL sodium selenite (Sigma-Aldrich). For subsequent cell cultures, 100 U/mL of rhIL-2, 5 ng/mL of rcIL-15 and 5 ng/mL of rcIL-21 were added to both media. After 7 days, rcIL-21 was excluded and only rhIL-2 and rcIL-15 were added for 28 days.

### Phenotyping of expanded canine NK cells by flow cytometry

To determine the phenotype and purity of canine NK cells expanded per week, cells were harvested, centrifuged, and washed with Fluorescence-Activated Cell Sorting (FACS) buffer (1% bovine serum albumin in phosphate buffered saline). Isolated cells were stained with various antibodies, including FITC-conjugated anti-dog CD3, APC-conjugated anti-dog CD5, PE-conjugated anti-dog CD8, PE-cy7 conjugated anti-dog CD4, PE-conjugated anti-dog CD21 (all from Bio Rad, USA), Dog Activated T Lymphocyte Cocktail (APC-conjugated anti-dog pan T cell marker; PE-conjugated anti-dog B cell marker; FITC-conjugated anti-dog T cell activation marker; BD Biosciences, USA), anti-dog TCRαβ (clone CA15.8G7), TCRγδ (clone CA20.8H1) (both from Peter Moor, UC Davis, USA), anti-canine NKp46 (Millipore, USA) (clone 48A), PE-conjugated Goat anti-Mouse IgG (H+L) Cross-Adsorbed Secondary Antibody, and pacific blue-conjugated anti-human CD14. These antibodies were added and incubated at 4°C for 15 min. Flow cytometry analysis was performed using a FACS canto II flow cytometer (Becton Dickinson). Data were analyzed with FlowJo software (FlowJo, LLC, USA); the gating strategy is shown in Supplementary Fig. 1.

### Measuring expanded canine NK cells purity and fold expansion

The predicted canine NK cells present in canine PBMCs are defined by the following phenotypes: CD5dimCD3+CD8+CD21−CD5−TCRαβ−TCRγδ− [22]. The purity of expanded canine NK cells was measured by phenotyping using a FACSCanto II flow cytometer (Becton Dickinson). The NK cell fold was determined by measuring the increase in NK cell purity from the initial culture (day 0) to the end of culture period (days 14, 21 and 28), accounting for cell divisions. Due to the lack of specific markers for canine NK cells and their changing phenotype in culture, non-B, non-T cells were used to define the purity of canine NK cells after day 0 [23].

### Real-time quantitative polymerase chain reaction

To investigate the differences in receptor expression based on the culture medium and feeder cells used, RNA was extracted from canine PBMCs and NK cells cultured for 2 weeks using TRIzol (Thermo Fisher, USA). cDNA was then synthesized from the extracted RNA using the ImProm-II™ Reverse Transcription System (Promega, USA). The expression levels of canine NK cell-associated genes, including beta-actin, granzyme B, perforin, NKp44, NKp30, IFN-γ, TNF-α, Ly49 and NKG2D were analyzed by quantitative reverse transcription polymerase chain reaction (qRT-PCR) using specific primers (Table 1), which was performed with TB Green Premix (Takara Bio, Japan) and the Rotor-Gene Q system (QIAGEN, Germany). All samples were assayed in triplicate. Relative target-gene mRNA levels were quantified using cycle threshold (Ct) values relative to β-actin. Data are reported as 2^−(Ct of target − Ct of β-actin)^ in arbitrary units.

**Table 1:** Oligonucleotide primers used for quantitative reverse transcription polymerase chain reaction of canine natural killer cell-associated genes

Gene (accession number)	Primer sequence	Size (bp)
Beta-actin (Z70044)	F: ACCAACTGGGACGACATGGAGA	205
	R: AGGCATACAGGGACAGGACAG	
Granzyme B (XM547752)	F: GGAGAGATCATCGGGGGACATGA	195
	R: CTCCTGTTCCTTGATGTTGTGG	
Perforin (FJ973622)	F: ACAGGTACAGCTTCAGCACTGAC	189
	R: CCATGGACCGGATGAAGTGGGT	
INFγ (FJ194478)	F: AGTAAGAGGGAGGACTTCC	174
	R: TTATTTCGATGCTCTGCGGC	
Ly49 (AY191818)	F: GATTCTTGGGATCCTGTGTTTACT	236
	R: GTAAATCCTGTTCCTTTTTCTGCTG	
NKG2D (XM849013)	F: GTTATTGTGGTCCGTGTCCTAA	222
	R: ACTGCCAGGATCCATTTGTTGG	
NKp30 (DQ003484)	F: CTTCTTGCCGTGTTCCTTCAA	236
	R: CCAGAACCTCCACTCTGCACA	
NKp44 (XM846203)	F: ACCAGGTTAGTCAGCAGCTCCA	205
	R: CTGGAGACACTGCCAGATAGAAT	

F, forward; R, reverse; bp, base pairs; qRT-PCR, quantitative reverse transcription polymerase chain reaction.

### Cytotoxicity of expanded canine NK cells against CTAC cells

Cytotoxicity of canine NK cells on day 14 of culture against CTAC cells was measured using CCK-8 (Biotool, USA) [24]. In an F-type 96-well plate, 1 × 10^4^ of CTAC cells were seeded, and 5 × 10^4^ of 2-week cultured canine NK cells were added (effector-to-target ratio; 5:1). The NK cells were co-cultured with the CTAC cells for 3 h in a 37°C incubator. Then, 10 μL of CCK-8 reagent was added to each well and incubated for another hour without mixing. After 1 h, the contents were mixed well and centrifuged at 1,500 rpm for 3 min. The 90 μL of the supernatant was then used to measure the absorbance at 450 nm (A450) using a SpectraMax M2 (Molecular Devices, USA). To avoid dye interference, all CCK-8 assays utilized phenol red–free media. The formula for calculating cytotoxicity using absorbance is as follows:

Cytotoxicity (%) = 100% − 100 × [A450 of Effector Cell-Treated Target Cells − A450 of Effector Cells (Background of Effector Cells)]/[A450 of Target Cells − A450 of Target Cells With No CCK-8 (Background of Target Cells)]

### Statistical analysis

PASW statistics 18 (SPSS Inc., USA) was used to determine differences between groups in purity, amplification rate, fold expansion and expression of receptors associated with expressing NK cells. Normality was assessed prior to analysis. Overall group differences were evaluated using the Kruskal-Wallis test, followed by pairwise Mann-Whitney *U* tests with Bonferroni correction. Statistical significance was set at *p* < 0.05.

## RESULTS

### Purity and expansion rate of expanded canine NK cells

**Fig. 1:**
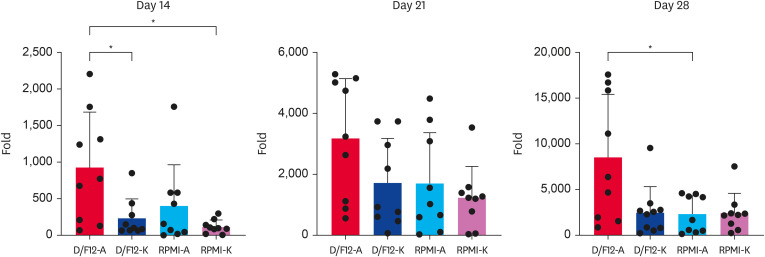
Fold expansion of canine NK cells following *ex vivo* culture. Canine NK cells were cultured for 28 days using D/F12 medium or RPMI medium with GE-ARH77 cells or GE-K562 cells (n = 9). NK, natural killer; D/F12, mixture of Dulbecco’s Modified Eagle Medium and Ham’s F-12 Nutrient; A, GE-ARH77; K, GE-K562. ^*^p < 0.05.

**Fig. 2:**
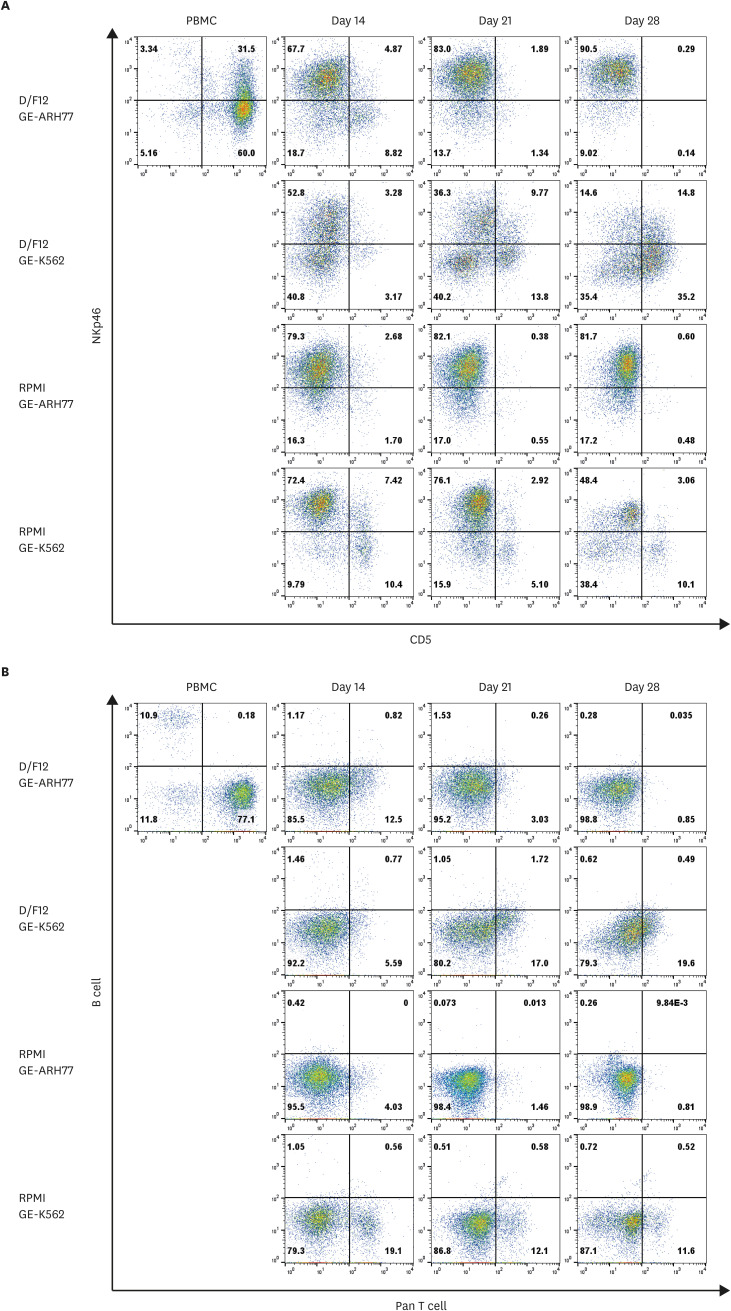
Phenotype of canine NK cells during culture for 28 days using D/F12 medium or RPMI medium with GE-ARH77 cells or GE-K562 cells. (A) CD5−NKp46+ cells. (B) Non-B, non-T cells (n = 5). NK, natural killer; D/F12, mixture of Dulbecco’s Modified Eagle Medium and Ham’s F-12 Nutrient; PBMC, peripheral blood mononuclear cell.

The effects of different media and feeder cells were assessed to identify an efficient method of expanding canine NK cells. Specifically, RPMI-1640 and DMEM/F12 media were compared in terms of their ability to support the expansion rate (Fig. 1) and purity (Fig. 2) of *ex vivo* expanded canine NK cells. Additionally, the expansion rate and purity of NK cells cultured with GE-K562 and GE-ARH77 feeder cells were assessed. Using DMEM/F12-based medium and GE-ARH77 cells, the results showed 87% (80.5%–92.7%) purity and 928.53 (67.8–2,205.5) expansion rate on day 14, 93% (78.7%–97.7%) purity and 3,177.77 (547.7–5,283.2) fold at day 21, and 97% (91.7%–99.6%) purity and 8,515.48 (870.6–17,564.1) expansion rate on day 28. With DMEM/F12 medium and GE-K562 cells, we observed 87% (78.7%–91.9%) purity and 232.93 (63.6–850.2) expansion rate on day 14, 86% (73.8%–95.9%) purity and 1,716.93 (58.3–3,739. 5) expansion rate on day 21, and 80% (40.0%–95.7%) purity and 2,468.97 (236.6–9,561.6) expansion rate on day 28. Using RPMI medium and GE-ARH77 cells, we found 92% (83.3%–97.8%) purity and 405.69 (3.4–1,758) expansion rate on day 14, 98% (96.4%–99.3%) purity and 1,703.68 (30.4–4,489) expansion rate at day 21, and 98% (95.3%–99.4%) purity and 2,301.92 (136.4–4,594) expansion rate at day 28. Finally, with RPMI medium and GE-K562 cells, the results showed 88% (74.2%–97.1%) purity and 116.45 (5.2–297.9) expansion rate at day 14, 92% (74.2%–99.1%) purity and 1,230.26 (38.7–3,533.3) expansion rate on day 21, and 97% (88.4%–99.7%) purity and 2,442.83 (220.3–7,549) expansion rate on day 28. Overall, DMEM/F12 medium and GE-ARH77 cells demonstrated the highest expansion rate and purity across at all time points. In DMEM/F12 medium, GE-ARH77 cells consistently exhibited significantly higher expansion rates than GE-K562 cells (*p* < 0.05). In RPMI medium, GE-ARH77 cells showed higher expansion rates at week 2, similar rates in week 3, and lower rates than GE-K562 cells in week 4. However, no significant differences were observed between the groups in the RPMI medium. These results suggest that GE-ARH77 cells and DMEM/F12 medium are more effective for expanding canine NK cells. In addition, *ex vivo* canine NK cell expansion from PBMCs was unsuccessful in some donors cultured in RPMI medium (Supplementary Fig. 2).

### Phenotype of expanded canine NK cells using flow cytometry

**Fig. 3:**
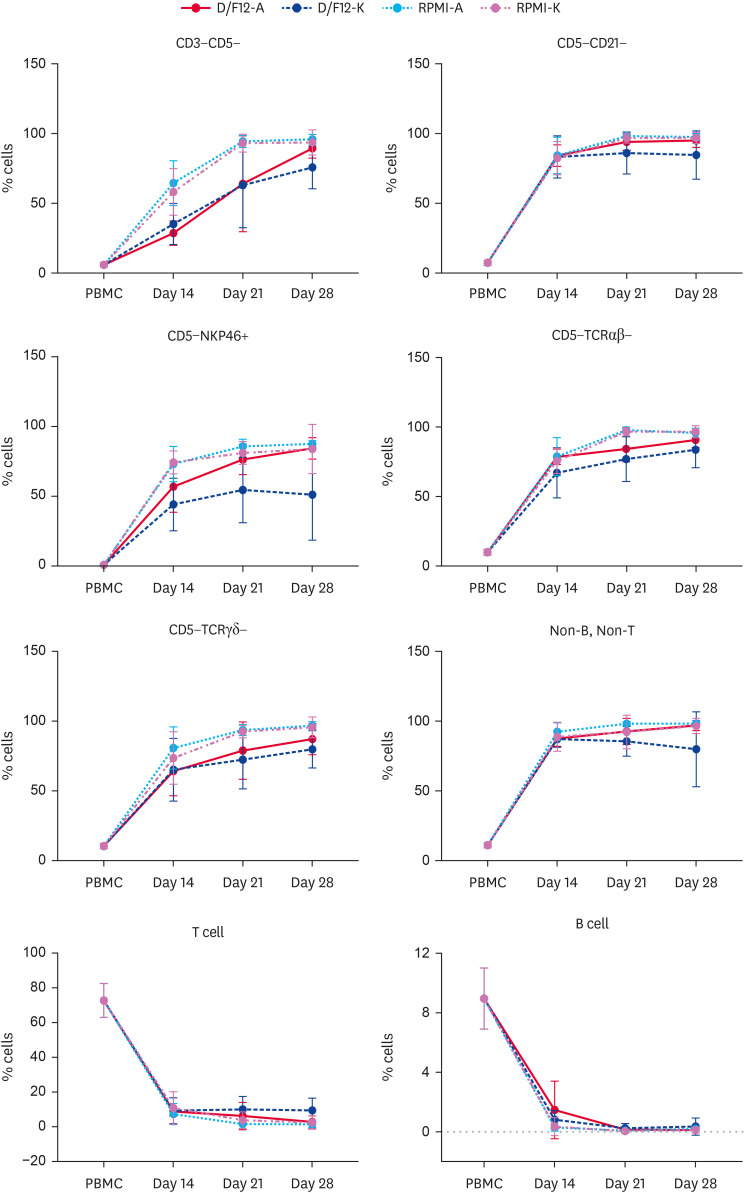
Phenotype of change of CD3−CD5−, CD5−CD21−, CD5−NKp46+, CD5−TCRαβ−, CD5−TCRγδ−, T cells, B cells, non-B, non-T cells during culture for 28 days using D/F12 medium or RPMI medium with GE-ARH77 cells or GE-K562 cells (n = 5). D/F12, mixture of Dulbecco’s Modified Eagle Medium and Ham’s F-12 Nutrient; A, GE-ARH77; K, GE-K562; PBMC, peripheral blood mononuclear cell.

Flow cytometry was used to analyze the phenotypic characteristics of canine NK cells throughout the expansion period. The expression of various surface markers was evaluated at different time points (PBMC, days 14, 21 and 28, respectively) under varying culture conditions by comparing both media and feeder cells (Figs. 2 and 3, Supplementary Fig. 3). Although both media types supported NK cell expansion, DMEM/F12 medium combined with GE-ARH77 cells consistently demonstrated the highest fold expansion of NK cells throughout the culture period (Fig. 1). Phenotypic analysis revealed consistent development of NK cell characteristics across all culture conditions, with some notable differences in marker expression patterns (Figs. 2 and 3). Initially, the proportion of CD3-CD5-cells in PBMCs was 5.96%. Under DMEM/F12 medium with GE-ARH77 cells, CD3−CD5− cells exhibited a gradual increase (28.7% and 64.06% on days 14 and 21, respectively), ultimately reaching 89.44% by day 28, demonstrating stable and continuous expansion (Fig. 3). The CD5−CD21− population demonstrated a particularly robust expansion in the DMEM/F12 medium with GE-ARH77 cells condition, increasing from 7.39% to 95.06% by day 28, comparable to that in the RPMI medium (Fig. 3). For T cell marker analysis, both TCRαβ and TCRγδ negative populations were evaluated (Fig. 3). The CD5−TCRαβ− population in the DMEM/F12 medium with GE-ARH77 cells group showed steady increase from 9.90% in PBMCs to 90.72% by day 28, while CD5−TCRγδ− cells reached 87.16%, indicating effective T cell exclusion during expansion. Notably, the NK cell-specific marker NKp46 showed significant expansion in the DMEM/F12 medium with GE-ARH77 cells (Figs. 2A and 3). The CD5−NKp46+ population increased from an initial 0.77% to 84.38% by day 28, indicating a robust NK cell development. This expansion pattern, combined with the highest fold expansion rate, suggested that DMEM/F12 medium with GE-ARH77 cells effectively supported both the quantity and quality of NK cell expansion (Fig. 1). The non-B and non-T cell population in the DMEM/F12 medium with GE-ARH77 cells showed excellent expansion from 11.05% to 96.90% by day 28, indicating efficient enrichment of the NK cell population while effectively excluding B and T cells (Fig. 2B and 3). While RPMI medium conditions showed higher percentage values for some markers, the superior fold expansion achieved with DMEM/F12 medium with GE-ARH77 cells suggested that this combination provides optimal conditions for *ex vivo* expansion of canine NK cells. This culture condition successfully supported both substantial numerical expansion and maintenance of the NK cell phenotype, making it particularly suitable for the clinical-scale production of canine NK cells.

### Expression of expanded canine NK cell-associated receptors

**Fig. 4:**
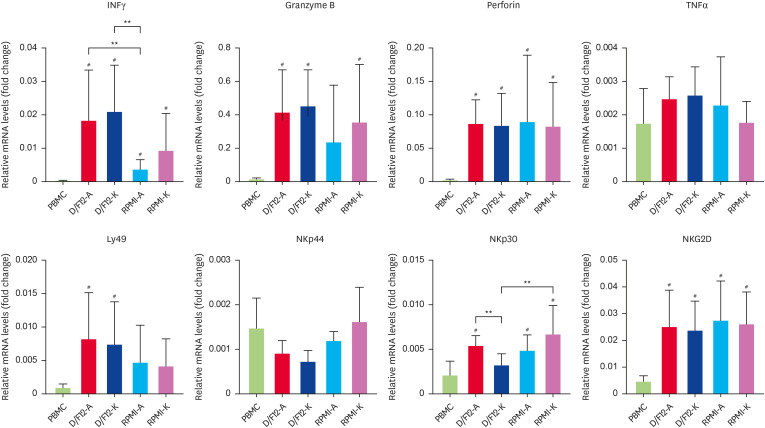
Expression of NK-associated and receptor and function of expanded canine NK cells using D/F12 medium or RPMI medium with GE-ARH77 cells or GE-K562 cells on day 14. mRNA expression levels were determined relative to beta actin (n = 5). NK, natural killer; D/F12, mixture of Dulbecco’s Modified Eagle Medium and Ham’s F-12 Nutrient; A, GE-ARH77; K, GE-K562; PBMC, peripheral blood mononuclear cell. ^**^p < 0.01, ^#^*p* < 0.05 vs. relative mRNA level of PBMCs.

The expression of NK cell-associated genes was analyzed in expanded NK cells on day 14 using qRT-PCR (Fig. 4). Gene expression levels were normalized to β-actin and presented as relative fold changes compared to that of PBMC expression levels.

Cytotoxicity-related genes were significantly upregulated in DMEM/F12 medium. IFN-γ expression was markedly increased in DMEM/F12 medium groups, as GE-ARH77 cells and GE-K562 cells showed 112.1-fold and 128.3-fold increases compared to PBMCs, respectively, in the medium. This was substantially higher than that in the RPMI medium (GE-ARH77: 23.0-fold, GE-K562: 56.9-fold, *p <* 0.05). Similarly, granzyme B expression was notably elevated in DMEM/F12 medium, with GE-ARH77 and GE-K562 cells showing a 41.3- and 45.1-fold increase compared to PBMCs, whereas the RPMI medium groups showed a lower increase (GE-ARH77 cells: 23.3-fold, GE-K562 cells: 35.3-fold). Among the NK cell receptors, Ly49 was significantly upregulated in D/F12, with GE-ARH77 cells showing a 10.0-fold increase compared to PBMCs, which was higher than that in the RPMI medium (GE-ARH77 cells: 5.7-fold, GE-K562 cells: 4.9-fold). The expression of natural cytotoxicity receptors exhibited varying patterns. NKp30 expression was enhanced in DMEM/F12 medium with GE-ARH77 cells (2.6-fold increase), whereas NKp44 showed slightly decreased expression across all conditions. NKG2D and Ly49 were consistently upregulated under all conditions. These results demonstrate that DMEM/F12 medium with GE-ARH77 cells as feeder cells effectively supports the expression of key NK cell-associated genes, particularly those involved in cytotoxicity (IFN-γ, granzyme B) and NK cell receptors (Ly49, NKG2D). This culture condition resulted in superior or comparable expression levels for most of the analyzed genes, suggesting its effectiveness in maintaining NK cell functionality during *ex vivo* expansion.

### Cytotoxicity of expanded canine NK cells against CTAC cells

**Fig. 5:**
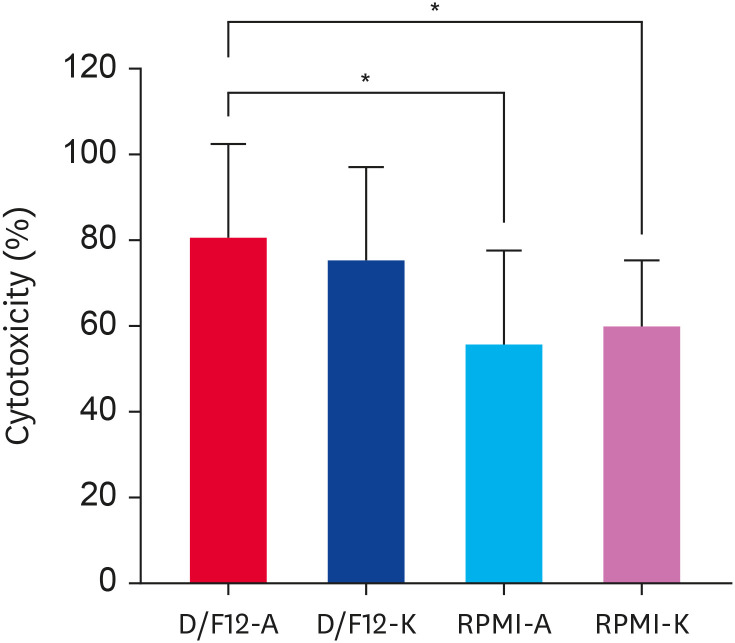
The cytotoxicity of expanded canine NK cells using D/F12 medium or RPMI medium with GE-ARH77 cells or GE-K562 cells at day 14 against CTAC cells. The cytotoxicity effects of NK cells were measured at 5:1 effector to target ratio (n = 8). NK, natural killer; D/F12, mixture of Dulbecco’s Modified Eagle Medium and Ham’s F-12 Nutrient; A, GE-ARH77; K, GE-K562; CTAC, canine thyroid adenocarcinoma. ^*^p < 0.05.

Cytotoxicity of expanded canine NK cells against CTAC cells under different culture conditions was evaluated by varying both medium types and feeder cells (Fig. 5). Canine NK cells expanded in DMEM/F12 medium demonstrated significantly higher cytotoxicity than those cultured in RPMI medium, regardless of the feeder cell type used. Specifically, NK cells expanded in DMEM/F12 medium with GE-ARH77 cells showed the highest cytotoxicity (80.69%) among the groups against CTAC cells, followed closely by those expanded in DMEM/F12 medium with GE-K562 cells (75.53%); however, no significant difference was observed. In contrast, NK cells expanded in RPMI medium showed relatively lower cytotoxic activity, with the GE-ARH77 and GE-K562 cells groups exhibiting 55.85% and 60.07% cytotoxicity, respectively. Statistical analysis revealed a significant difference in cytotoxicity between the DMEM/F12 and RPMI medium groups was significant (*p* < 0.05), whereas no significant differences were observed between GE-ARH77 cells and GE-K562 cells as feeder cells within the same media group. These results suggest that DMEM/F12 medium provides superior conditions for generating functionally active NK cells with enhanced cytotoxic capacity, independent of the feeder cell type used for expansion.

## DISCUSSION

This study aimed to evaluate and provide comprehensive insights into the optimization of *ex vivo* expansion conditions for canine NK cells by comparing different media types and feeder cells. It was determined that the combination of DMEM/F12 medium with GE-ARH77 feeder cells provides optimal conditions for canine NK cell expansion, resulting in up to 8,515-fold increases by day 28 with high purity (> 97%). However, no significant differences in cytotoxicity were observed between feeder types, indicating that enhanced expansion does not necessarily lead to improved cytotoxic function. A key finding of our study was the superior performance of DMEM/F12 medium compared to that of RPMI medium, particularly when combined with GE-ARH77 feeder cells. Notably, in some donors, *ex vivo* canine NK cell expansion from PBMCs was unsuccessful in RPMI medium, suggesting that this medium may not be optimal for canine NK cell expansion protocols (Supplementary Fig. 2). This combination consistently resulted in higher expansion rates and enhanced cytotoxicity against CTAC cells. The superior performance of DMEM/F12 medium may be attributed to its richer nutrient composition and better support of cellular metabolism, as previously observed in other immune cell culture systems [13,25]. Notably, the DMEM/F12 condition also contained β-mercaptoethanol, GlutaMAX, and selenium, which support redox balance and glutamine availability and can augment NK-cell proliferation and function; therefore, the observed advantage likely reflects the combined effects of the base medium and these supplements, not the basal formulation alone [13]. Additionally, interactions with the engineered feeder cells—via cytokine delivery and costimulatory signaling—may synergize with this supplement profile to further enhance proliferation and cytotoxicity, indicating a media–supplement–feeder coupling rather than a medium-only effect [4,5,25]. Phenotypic analysis revealed a significant enrichment of canine NK cell populations by day 28 in the DMEM/F12 medium with GE-ARH77 cells. This robust expansion of the NK cell population, coupled with the effective exclusion of B and T cells, suggests that our culture conditions successfully maintained NK cell lineage commitment while preventing unwanted lymphocyte expansion [6,26,27]. Cytotoxic-related genes were substantially upregulated in DMEM/F12 medium. The dramatic increase in IFN-γ expression and granzyme B suggests enhanced cytotoxic potential of the expanded NK cells. These findings align with those of previous studies, demonstrating the importance of these molecules in NK cell-mediated tumor cell killing [28,29]. The differential expression patterns of NK cell receptors under various culture conditions provide valuable insights for the optimization of NK cell functionality. The enhanced expression of Ly49 and the maintained expression of NKG2D in DMEM/F12 with GE-ARH77 cells suggested a preserved NK cell receptor repertoire, which is crucial for target cell recognition and killing [30]. Our findings have important implications for both research and clinical applications. The demonstrated superiority of DMEM/F12 medium with GE-ARH77 cells provides a robust platform for generating large numbers of functional canine NK cells, which could be particularly valuable for adoptive immunotherapy applications. In conclusion, our study establishes DMEM/F12 medium with GE-ARH77 feeder cells as an optimal combination for *ex vivo* expansion of canine NK cells, providing both superior expansion rates and functional capabilities. These findings contribute significantly to the advancement of NK cell–based immunotherapy in veterinary medicine and provide a foundation for future optimization studies. Clinically, NK platforms show a favorable safety profile with low rates of cytokine release syndrome, neurotoxicity, and graft-versus-host disease and demonstrate *in vivo* persistence—particularly when engineered with IL-15—supporting therapeutic potential and repeat dosing. In veterinary contexts, first-in-dog studies indicate feasibility and acceptable safety, and adjunctive radiotherapy has been shown to enhance NK homing and cytotoxicity in canine sarcomas, reinforcing translational credibility and combination-therapy rationale [31,32,33]. Collectively, our results support the translational trajectory of NK cell–based immunotherapy in veterinary oncology and outline concrete next steps toward clinical application.

## SUPPLEMENTARY MATERIALS

Supplementary Fig. 1 Gating strategy for the identification of canine natural killer cells and other lymphocyte subsets including isotype controls.

Supplementary Fig. 2 Donor-dependent variability in *ex vivo* expansion of canine natural killer cells across different base media conditions.

Supplementary Fig. 3 Phenotype of change of CD3−CD5−, CD5−CD21−, CD5−NKp46+, CD5−TCRαβ−, CD5−TCRγδ− cells during culture for 28 days using D/F12 medium with GE-ARH77 cells (A), DMEM/F12 with GE-K562 (B), RPMI medium with GE-ARH77 (C), RPMI medium with GE-K562 cells (D) on donor 1.
